# Development of Wide-Angle Depolarizing Reflector at 1064 nm

**DOI:** 10.3390/ma16124258

**Published:** 2023-06-08

**Authors:** Han Zhu, Hongyan Jiang, Kai Guo, Yongchao Peng, Yawu Xin, Gong Zhang, Yixin Lin, Ning Yang, Huashu Wei, Zekai Huang, Shifu Xiong, Zhanggui Hu

**Affiliations:** 1 Tianjin Key Laboratory of Functional Crystal Materials, Institute of Functional Crystals, Tianjin University of Technology, Tianjin 300384, China; zh_optics@stud.tjut.edu.cn (H.Z.); pycoptical@stud.tjut.edu.cn (Y.P.); xyw_optics@stud.tjut.edu.cn (Y.X.); linyixin@stud.tjut.edu.cn (Y.L.); 20200988yangning@stud.tjut.edu.cn (N.Y.); jingyuan20204495@stud.tjut.edu.cn (H.W.); apple0577@stud.tjut.edu.cn (Z.H.); hu@mail.ipc.ac.cn (Z.H.); 2 National Key Laboratory of Science and Technology on Tunable Laser, Harbin Institute of Technology, Harbin 150080, China; jhy_optics@stu.hit.edu.cn; 3 Optoelectric Component Research and Development Center, Beijing Institute of Control Engineering, Beijing 100094, China; kg_optics@126.com; 4 Department of Optics and Electric Engineering, Changchun University of Science and Technology, Changchun 130022, China; gongzhang@cust.edu.cn

**Keywords:** optical thin film, optical coherence tomography, wide-angle, depolarized

## Abstract

Optical coherence tomography is a new promising chromatographic imaging technique with the advantages of noncontact and high resolution without damage, which is widely used in the field of biological tissue detection and imaging. As an important optical element in the system, the wide-angle depolarizing reflector plays a key role in the accurate acquisition of optical signals. Ta_2_O_5_ and SiO_2_ are selected as the coating materials for the technical parameter requirements of the reflector in the system. Based on the basic theory of optical thin film and combined with MATLAB and OptiLayer software, the design of 0~60° incident 1064 ± 40 nm depolarizing reflective film is realized by establishing the evaluation function of the film system. To optimize the oxygen-charging distribution scheme during film deposition, the weak absorption properties of the film materials are characterized by optical thermal co-circuit interferometry. According to the sensitivity distribution of the film layer, the optical control monitoring scheme with a thickness error of less than 1% is designed rationally. “Crystal control + optical control” is used to precisely control the thickness of each film layer and complete the preparation of resonant cavity film. The measurement results show that the average reflectance is more than 99.5%, and the deviation of P-light and S-light is less than 1% in the 1064 ± 40 nm wavelength band range from 0° to 60°, which meets the requirements of optical coherence tomography system.

## 1. Introduction

Optical coherence tomography (OCT) is an imaging technique that uses the basic principle of weak coherent light interferometry to detect the backward reflection or several scattering signals of incident weak coherent light at different depth levels of biological tissues and obtain two-dimensional or three-dimensional structural images of biological tissues. It has the advantages of being noncontact and high-resolution and has a wide range of applications in medical imaging and industrial inspections [1,2,3]. High-quality wide-angle depolarizing reflectors as key components in optical coherence tomography systems are the main factors affecting the imaging quality of the system for the accurate acquisition of optical signals [4].

**Figure 1:**
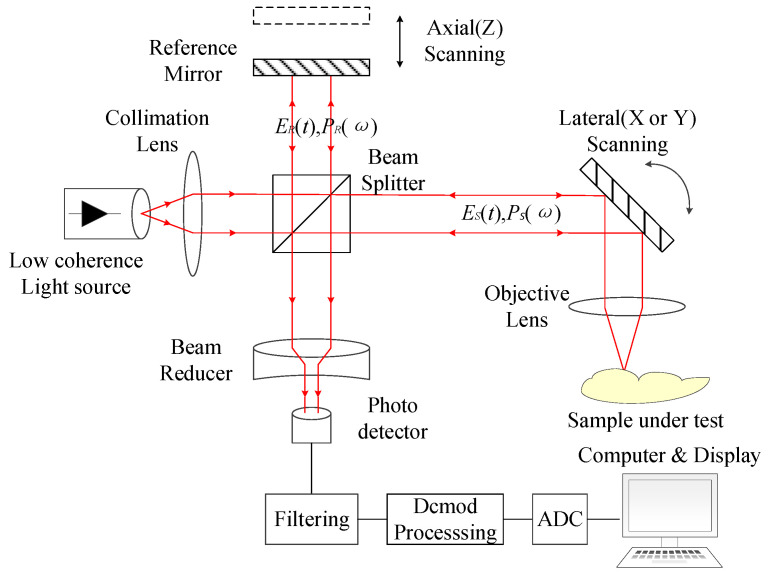
Schematic diagram of optical coherence tomography system working principle [6].

The schematic diagram of the optical coherence tomography system is shown in Figure 1. The light emitted by a beam near-infrared light source is split in two. A beam of light is irradiated onto the tested object (vascular tissue) called the signal arm, and the other beam is incident to the reference reflector called the reference arm. The light signal reflected back from the object to be measured shows different strengths and weaknesses with its shape and is overlaid with the reference light signal reflected from the reflector. When the lengths of the signal arm and the reference arm are the same, interference occurs. The signal is enhanced when the light wave fixed position is in the same direction and weakened when the light wave fixed position is in the opposite direction. With the mobilization of the reflector position, the length of the reference arm is changed, and the signals of different depths of tissues are obtained, and these light signals can be processed by a computer to obtain tissue tomography images [5]. In order to improve the signal strength and system stability of the optical coherence tomography system, a wide-angle depolarizing reflector needs to be developed. BK7 (n = 1.521) colorless optical glass was selected as the substrate with the characteristics of no bubbles, high transmittance, and high resistance to acid and alkali. The reflector needs to meet the average reflectance of more than 99.5% and the deviation of P-light and S-light of less than 1% in the 1064 ± 40 nm wavelength band range from 0° to 60°. This presents a great challenge for the design and development of thin films. 

According to relevant research, in 2007, Wenliang Wang et al. used the needle-thin film synthesis method and conjugate graduate refinement method and chose the materials and structures of the initial film system based on Costich and Thelen’s theory to design three-wavelength depolarizing parallel-plate light-splitting film of 532, 633, and 1315 nm [7]. In 2013, Xiaodan Gao completed the design of variable-angle 3~5 μm IR depolarization and transmission enhancement film, and the incidence of angle with 45° light-splitting film from 1260 nm to 1360 nm spectral range was designed by resolution, needle, and variable metric optimization methods using the initial formula designed by TFCalc formula design software [8,9]. In 2015, Xiuhua Fu et al. used a combined metal-dielectric structure to design a 500~900 nm spectrophotometric film to solve the polarization separation problem arising from the tilted incidence of a broad-spectrum beam [10]. In 2016, Qiuhui Zhuang et al. designed and developed a depolarization spectrophotometer with an operating band of 0.35~1.7 μm, and the optical efficiencies of the reflection and transmission band reached 97% and 91%, respectively, with the polarization sensitivity of the reflection band controlled within 1.5% [11]. However, for the wide-angle (0~60°) depolarizing reflective film, no relevant reports have been found. The wide-angle depolarizing reflector studied in this work needs to have high reflectivity in the given angle and wavelength range and the deviation of P and S light at an oblique incidence of less than 1%, which brings great challenges to the design and preparation of the film system and places higher requirements on the film system design, film preparation process, and the control accuracy of the film thickness.

## 2. Experiments

### 2.1. Materials Selection Analysis

The coating materials for the transparent region in the near-infrared (NIR) band are selected according to the technical parameter requirements shown in Table 1, and factors, such as material refractive index, mechanical properties, film layer stress matching, and film stability, are considered [12,13]. In the NIR band, the common coating materials are TiO_2_, ZrO_2_, HfO_2_, Ta_2_O_5_, MgF_2_, and SiO_2_. The characteristics of the materials are shown in Table 2.

TiO_2_, ZrO_2_, HfO_2_, and Ta_2_O_5_ are commonly used as high-refractive-index materials in near-infrared bands. TiO_2_ has a higher refractive index but is prone to oxygen vacancy defects during film formation and generates multivalent oxides due to changes in oxygen charge; the optical constants are greatly affected by the coating environment [14]. For the melting point to be higher than the sublimation point, ZrO_2_ material is directly deposited from the solid phase to the gas phase on the substrate, so there are problems such as unstable evaporation rate and low laser damage threshold [15]. HfO_2_ film has the characteristics of low absorption and high damage threshold, but phase transformation often occurs during evaporation with the sputtering phenomenon, and nodular defects are easily formed on the surface of the film [16]. Ta_2_O_5_ has the property of a high damage threshold, low absorption, stable physical and chemical properties, etc. According to the relevant literature, Ta_2_O_5_ film has a more stable saturated Ta-O bond structure and possesses a longer structural delay time than HfO_2_ films under 1064 nm laser radiation, i.e., they are less prone to phase transformation under laser radiation [17]. On balance, Ta_2_O_5_ was chosen as a high refractive index material.

The common low-refractive-index materials are MgF_2_ and SiO_2_. The MgF_2_ films deposited at high temperatures are hard and durable but prone to sputtering due to the high tensile stress. The film layer is prone to cracking when the accumulated thickness exceeds 1.4 μm [18]. SiO_2_ films have stable performance, high laser damage threshold, and low absorption loss, and belong to the oxide such as Ta_2_O_5_, which can effectively reduce or avoid the negative effects of oxygen charging in the preparation of Ta_2_O_5_ films. The two materials are well matched [12,19]; therefore, SiO_2_ is chosen as the low-refractive-index coating material.

### 2.2. Materials and Software

Ta_2_O_5_ (99.998%, JuBo Guneng (Suzhou, China) Thin Film Materials Co., Ltd.), SiO_2_ (99.9997%, JuBo Guneng (Suzhou) Thin Film Materials Co., Ltd.).

OptiLayer (13.77, OptiLayer GmbH, Ismaning, Germany) software was used to design the film system and analyze the sensitivity of each film layer. MCalc (V4.0.21, Leybold Optics (Beijing, China) Co., Ltd.) software was used to calculate the optical constants of the films and design the optical control monitoring scheme.

### 2.3. Preparation of Thin Film

The experiment was completed on the Leybold SYRUSpro1110 vacuum coater, which is equipped with dual “e-type” electron guns, dual resistance evaporators, dual molecular pumps, Polycold deep cooling system, APSpro ion source, plasma ceramic plate heating system, OMS5100 optical film thickness monitoring system (four-position chip changing mechanism, monitoring wavelength range of 360~1750 nm), and six-probe crystal control system.

Before deposition, the vacuum chamber needed to be cleaned, the optical control chip and quartz crystal control chip needed to be replaced, and Ta_2_O_5_ and SiO_2_ film materials needed to be added. The substrate was scrubbed with a mixture of anhydrous ether and anhydrous ethanol in a 3:1 volume ratio, placed on the workpiece tray, and vacuumed with a proper speed. When the vacuum level reached 8.0 × 10^−5^ mbar, the baking was opened, and heating was initiated. After reaching the set temperature of 180 °C for 30 min, the ion source was turned on to clean the substrate for 120 s. When the vacuum reached 1.0 × 10^−5^ mbar, the evaporation plating process began. The actual plating process parameters are shown in Table 3.

Electron beam evaporation is an extremely complex process of evaporating solid material and condensing to form a thin film on the substrate surface through gas phase transport, which is affected by temperature, vacuum, evaporation rate, and oxygenation [20]. Generally speaking, the higher the substrate temperature, vacuum, and evaporation rate, the higher the kinetic energy that the evaporated film material can obtain, which can fully diffuse on the substrate surface and increase the film aggregation density and, thus, increase the refractive index [21]. However, as the temperature continues to rise, the film will have large residual stress, and if the evaporation rate is too high, it will increase the surface roughness of the film and affect the film thickness control accuracy. Under these conditions, the deposition rates of Ta_2_O_5_ and SiO_2_ were 0.3 nm/s and 0.7 nm/s, respectively. The oxygen charge during the deposition of the films was investigated.

Oxide materials, such as Ta_2_O_5_ and SiO_2_, have, to some degree, oxygen loss phenomenon during the deposition process, resulting in a certain absorption of the film, which not only affects the optical quality of the film but also causes damage to the laser film [22]. In order to solve this problem, it is usually required to fill the vacuum chamber with a certain amount of O_2_, during the film deposition. We have two O_2_ air intakes at the ion source (APS) and the electron gun (HPE), respectively, in the vacuum coater, which affect the tantalum oxide film differently. The oxygen charged from the electron gun takes the form of oxygen molecules, while the oxygen charged from the ion source is ionized into oxygen ions. Therefore, the effect of oxygen charge distribution on the absorption loss of the film is a more complex process. In order to maintain a constant vacuum level during film deposition, the total oxygen-charging flow rate is set to 50 sccm, and the oxygen-charging distribution scheme is shown in Table 4.

### 2.4. Characterization

The weak absorption of thin films was measured by a photothermal weak absorption meter PCI-03 manufactured by Stanford photothermal solutions (SPTS), Pahoa, HI, USA. The reflectance test was performed on the experimental samples using a Cary 7000 spectrophotometer manufactured by Agilent, Selangor, Malaysia.

## 3. Results and Discussion

### 3.1. Study on Meonolayer Film

**Figure 2:**
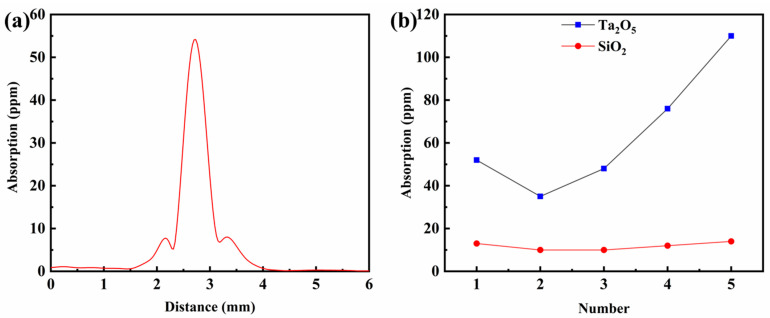
(**a**) Weak absorption test curve of 500 nm Ta_2_O_5_ monolayer film when oxygen flow rate at ion source is 15 sccm and oxygen flow rate at electron gun is 35 sccm. (**b**) Weak absorption test results of Ta_2_O_5_ and SiO_2_ monolayer films under different oxygenation schemes.

The weak absorption of thin films was measured by a photothermal weak absorption meter PCI-03 manufactured by SPTS [23]. Figure S1 shows the working principle of the self-built photothermal co-routing technology. The experimental pump laser wavelength is 1064 nm with a maximum output power of 10 W, which is a stronger beam, and there is a larger thermal absorption when it passes through the sample. The detection light wavelength is 633 nm with a power of about 1 mW, which is a weak beam and there is no thermal absorption when it passes through the sample. When the oxygen flow rate at the ion source is 5 sccm, and the oxygen flow rate at the electron gun is 45 sccm, the 500 nm weak absorption test result of Ta_2_O_5_ film is shown in Figure 2a, which is about 52 ppm, and this shape is typical of the weak absorption test graph of optical films.

According to the oxygenation distribution scheme in Table 2, the 500 nm Ta_2_O_5_ and SiO_2_ films were plated on the surface of fused silica glass, respectively, and characterized by weak absorptiometry under the same environmental conditions, and the results are shown in Figure 2b. It can be seen that as the oxygen-charging flow rate at the ion source increases from 5 sccm to 45 sccm and the oxygen-charging volume at the electron gun decreases from 45 sccm to 5 sccm, the weak absorption value of the Ta_2_O_5_ film decreases from 52 ppm to 35 ppm and then increases to 110 ppm. The reason for this phenomenon may be that the oxygen charged at the electron gun has a greater effect on the degree of oxidation of tantalum, while the oxygen charged at the ion source is ionized into oxygen ions with greater kinetic energy, which mainly affects the film aggregation density and refractive index. In the second oxygenation scheme, the tantalum oxide film has a minimum absorption value of 35 ppm, so a flow rate of 35 sccm at the electron gun and 15 sccm at the ion source is set for the deposited Ta_2_O_5_ film.

**Figure 3:**
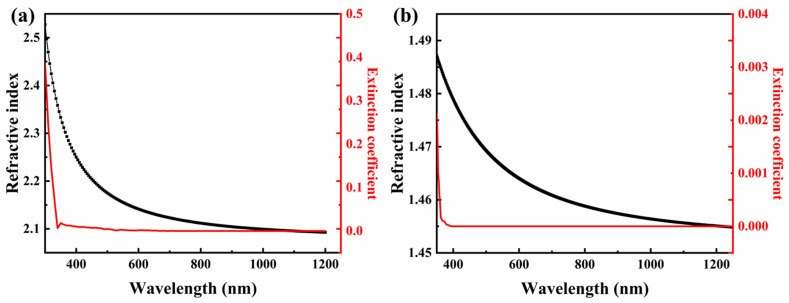
Refractive index and extinction coefficient of (**a**) Ta_2_O_5_ and (**b**) SiO_2_ films when the oxygenation flow rate at electron gun is 35 sccm and the flow rate at ion source is 15 sccm.

In addition, the weak absorption values of SiO_2_ films were generally lower than those of Ta_2_O_5_ films, and kept at about 10 ppm of different oxygenation distribution methods, indicating that the SiO_2_ films were deposited with basically no oxygen loss. Considering the ion source-assisted process, the 2nd oxygenation scheme was selected, i.e., the flow rate at the electron gun was set to 35 sccm and the oxygenation flow rate at the ion source was 15 sccm for the deposited Ta_2_O_5_ films. The optical constants of the films were fitted using MCalc software, and the results are shown in Figure 3. The optical constants obtained are used for the theoretical design of multilayer films.

### 3.2. Theoretical Design of Multilayer Optical Coatings

According to the optical thin film theory, for a multilayer film, when the number of layers is *k* (*k* = 1, 2, 3, …), the characteristic matrix of the combination of the membrane stack and the substrate is

(1) $$\left[\begin{array}{c} B\\ C \end{array}\right]=\left\{\prod_{j=1}^{k}\left[\begin{array}{cc} \cos\delta_{j} & i\sin\delta_{j}/\eta_{j}\\ i\eta_{j}\sin\delta_{j} & \cos\delta_{j} \end{array}\right]\right\}\left[\begin{array}{c} 1\\ \eta_{s} \end{array}\right]$$

where the potential phase thickness of the *j*th layer of the film is

(2) $$\delta_{j}=\frac{2\pi }{\lambda }N_{j}d_{j}\cos\theta_{j}$$

The combined conductance of the thin film system and substrate is

(3) $$Y=\frac{C}{B}$$

The reflectivity of the films is

(4) $$R=\left(\frac{\eta_{0}-Y}{\eta_{0}+Y}\right){\left(\frac{\eta_{0}-Y}{\eta_{0}+Y}\right)}^{*}$$

For normal incident light, the effective refractive index of the P and S components is the actual refractive index of the film. However, when the light is incident at an inclination, the effective refractive index of the P component is $\eta_{P}=n/\cos\theta$ and the effective refractive index of the S component is $\eta_{S}=n\cos\theta$, which will lead to different transmittance or reflectance of the two components and cause polarization separation. This polarization effect will cause some interference in the imaging of the optical coherence tomography system, so the polarization effect of the film should be minimized when the film system is designed.

For the wide-angle high-reflective film in this paper, Sub|(HL)^s H|Air is used as the base film system, where H represents Ta_2_O_5,_ the high-refractive-index material, and L represents SiO_2_ the low-refractive-index material, s represents the number of periods, and Sub represents the BK7 substrate. For the two selected materials, the reflection bandwidth can be determined by Equation (5).

(5) $$\Delta g=\frac{2}{\pi }\arcsin\frac{n_{H}-n_{L}}{n_{H}+n_{L}}.$$

where Δg, $n_{H}$ and $n_{L}$ are the relative wave numbers and refractive indices of high- and low-refractive-index materials, respectively.

For the wide-angle depolarized high-reflective film, it is difficult to design a film system with P and S light deviation of less than ±1% using the conventional evaluation function, so a new evaluation function is considered to be introduced. In the range of wavelength $\lambda_{1}$~$\lambda_{2}$ and angle $\theta_{1}$~$\theta_{2}$, when the difference between the reflectance $R_{j}(\lambda ,\theta )$ of *j*-layer film and the given ideal energy reflectance $R_{0}(\lambda ,\theta )$ is the minimum, the optical thickness of each layer is the ideal thickness, and the evaluation function is defined as

(6) $$f=\int_{\lambda_{1}}^{\lambda_{2}}\int_{\theta_{1}}^{\theta_{2}}\omega_{j}(\lambda ,\theta ){\left[{\left(R_{jP}(\lambda ,\theta )-R_{0}(\lambda ,\theta )\right)}^{2}+{\left(R_{jS}(\lambda ,\theta )-R_{0}(\lambda ,\theta )\right)}^{2}\right]}^{1/2}d\theta d\lambda$$

where $\omega_{j}(\lambda ,\theta )$ is the weighting factor, which depends on the light source energy distribution, $R_{jP}(\lambda ,\theta )$ is the *j*-layer film P light reflectance function, $R_{jS}(\lambda ,\theta )$ is the *j*-layer film S light reflectance function, $R_{0}(\lambda ,\theta )$ is the target amplitude reflectance function, and $R_{0}(\lambda ,\theta )=100$, $\omega_{j}(\lambda ,\theta )\equiv C$ (constant) in 1064 ± 40 nm band, 0~60° angle range in this paper.

**Figure 4:**
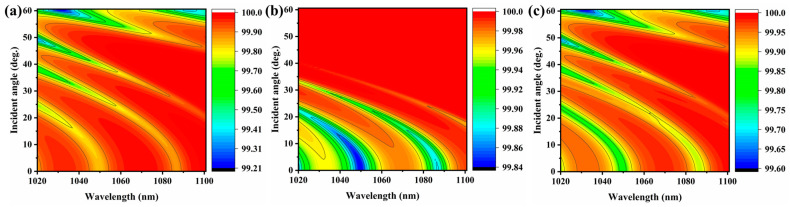
Theoretical calculated (**a**) P-light, (**b**) S-light, and (**c**) average reflectance spectral curve of multilayer optical coatings.

Equation (6) is imported into the genetic algorithm toolbox in MATLAB software, and the number of membrane layers of the thin film system is set to the number of variables, and the geometric thickness of each layer is limited to 50~500 nm so that the program is automatically optimized according to the target value. The optimal state is sought by continuous iterative calculations to reduce the difference between the theoretical and target values of the membrane system. After the calculation of the membrane system, the minimal value of the evaluation function is 0.137, and the optimized membrane system is Sub|1.030H 1.018L 1.058H 1.002L 1.041H 1.348L 1.117H 1.185L 1.065H|Air, with 55 layers and physical thickness of about 9.41 μm, where Sub is BK7 glass, H is Ta_2_O_5_ with an optical thickness of 1/4 wavelength, L is SiO_2_ with an optical thickness of 1/4 wavelength. Table S1 shows the wide-angle depolarization reflection film and the physical thickness of each film layer. Its theoretical design spectral curve is shown in Figure 4, with the average reflectance ${\bar{R}}_{ave}=99.9404\%$ at 0° incidence, the average reflectance ${\bar{R}}_{ave}=99.9618\%,R_{P}=99.9320\%,R_{S}=99.9915\%$ at 30° incidence, and the average reflectance ${\bar{R}}_{ave}=99.7927\%,R_{P}=99.5854\%,R_{S}=100\%$ at 60° incidence. It can be visualized from the two-dimensional diagram in Figure 4 that the film system obtained in the new evaluation function meets the design requirements in a wide range of angles and wavelengths, and the separation of P- and S-light polarization is less than ±1%.

### 3.3. Analysis of Optical Control Solutions

**Figure 5:**
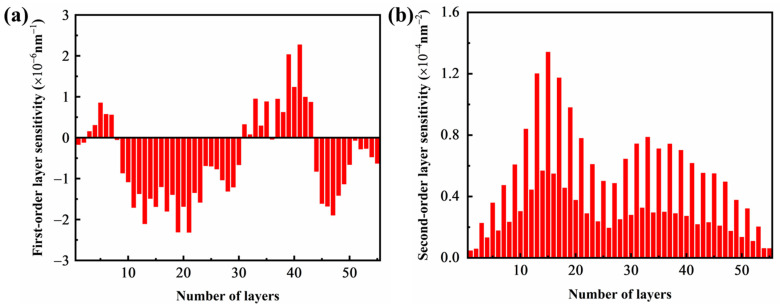
(**a**) First-order and (**b**) second-order sensitivity distribution diagram of multilayer optical films.

OptiLayer software was used to analyze the sensitivity of each film layer of the film system, as shown in Figure 5. The thickness control error exists in the film preparation process, and the effect of the error is not obvious for the whole film system when it occurs in the low-sensitivity layer. However, it will have a significant impact on the spectral properties of the film if it occurs in the higher-sensitivity layer, so it is necessary to control the high-sensitivity layer precisely to obtain better results, which is consistent with the theoretical design [24].

At present, there are mainly three kinds of film thickness control: time control, crystal control method, and optical control method [25]. Time control is based on the film deposition rate and total thickness to calculate the time required for deposition, which is mainly used in cases of extremely stable deposition rate, such as magnetron sputtering and ion beam sputtering methods to prepare thin film [26]. A crystal control method is used to monitor the physical thickness of the film by monitoring the amount of vibration frequency change of the AT-cut quartz crystal. The monitoring signal is linear, and it can detect the film deposition rate in real time [27]. The use of the optical control method allows for obtaining the optical thickness of the film by inversion of the light intensity change or polarization state change of the transmitted or reflected light due to film interference and has a compensation mechanism for film thickness errors. However, the signal varies sinusoidally with time, and it is difficult to monitor the film deposition rate [28].

During thin film deposition, due to the influence of temperature, vacuum, deposition rate, and other factors, each layer of film preparation has certain differences with the theoretical design, and different monitoring methods have advantages and disadvantages. Therefore, a reasonable combination of multiple monitoring methods can significantly improve monitoring accuracy. According to the equipment configuration, the film thickness is controlled by a combination of “crystal control + optical control”, the film deposition rate is controlled by the quartz crystal oscillation method, and the film thickness is monitored by the optical method.

The design is imported into the MCalc software, and the number of optical control pieces, monitoring wavelength, and other parameters is optimized to determine the optimum monitoring scheme. The number of optical controllers should be as few as possible in the premise of meeting the monitoring requirements to reduce the accumulation of residual errors caused by switching optical controllers during the film deposition process. Monitoring wavelength has the same or similar goal of reducing, as much as possible, the system errors introduced by the optical control system grating adjustment process.

**Figure 6:**
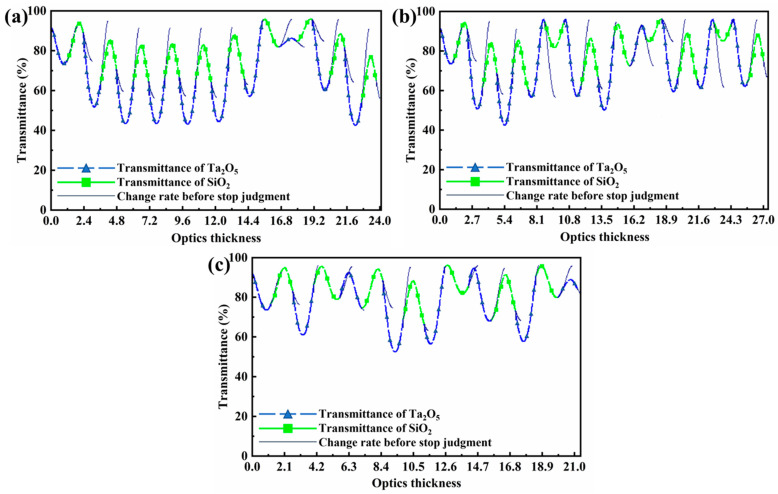
Optical monitoring design of wide-angle depolarized reflector (**a**) 1^#^, (**b**) 2^#^, and (**c**) 3^#^.

After comparative analysis, when the number of optical control pieces is lower than 3, it is impossible to find a monitoring scheme where the error of each layer can be controlled at 1%, so the number of optical control pieces in the monitoring scheme is determined to be 3. The 1st to 20th, 21st to 40th, and 41st to 55th layers are each monitored by one optical control piece, respectively, and each monitoring wavelength is 900 nm, and the optical signal changes with the thickness as shown in Figure 6, which shows that the scheme is reasonable and feasible. After the plating is completed, the samples are removed after the temperature of the samples is lowered to below 60 °C.

### 3.4. Analysis of Reflection Spectrum

**Figure 7:**
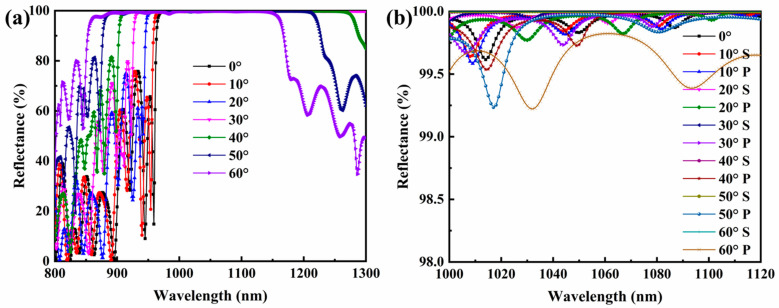
(**a**) Measured reflectance spectral curve of multilayer optical films. (**b**) P-light and S-light polarization separation comparison.

The reflectance test is performed on the experimental samples using a Cary 7000 spectrophotometer manufactured by Agilent, and the average reflectance spectral curves are shown in Figure 7a, and the P-light and S-light reflectance spectral curves are shown in Figure 7b. From the results, it can be seen that the average reflectance is greater than 99.5%, and the deviation of P-light and S-light is less than 1% in the range of 1064 ± 40 nm band and 0~60° wide angle, which meets the requirements for the use of the optical coherence tomography system.

## 4. Conclusions

In this paper, we focus on the study of the wide-angle depolarizing reflector in optical coherence tomography systems. Based on the optical thin film design theory and technical parameters, a new evaluation function for the film system design is established, and the design of the highly reflective film is realized by combining MATLAB and OptiLayer software. The influence law of oxygen-charging rate on the film quality was analyzed by the photothermal co-circulation absorption technique, and the oxygen-charging distribution scheme at the electron gun and ion source was determined. The film thickness monitoring scheme was optimized in the film plating process, and the experiment was conducted using vacuum thermal evaporation technology. A depolarized highly reflective film in the 1064 ± 40 nm band at 0~60° incidence, which meets the system usage requirements, is finally developed. With the rapid development of optical coherence tomography technology, the direction of future research work will be how to further reduce the P-light and S-light deviation at large angle incidence.

## Figures and Tables

**Table 1:** Technical parameters of wide-angle depolarizing near-infrared reflector.

Parameter	Specification
Substrate	BK7
Angle of incidence/(°)	0~60
Spectrum range/nm	1064 ± 40
Reflectivity/%	≥99
|Rp − Rs|/%	≤1

**Table 2:** Comparison of the properties of the commonly used coating materials in the NIR band.

Materials	Melting Temperature (°C)	Evaporating Temperature (°C)	Density (g/cm^3^)	Refractive Index	Transparent Region (μm)	Stress
TiO_2_	1850	2000	4.29	2.35	0.4~10	-
ZrO_2_	2715	2700	5.49	2.05	0.3~12	tensile stress
HfO_2_	2812	2700	9.68	2.00	0.22~12	-
Ta_2_O_5_	1800	2100	8.74	2.16	0.35~10	-
MgF_2_	1266	1540	2.90	1.38	0.11~10	tensile stress
SiO_2_	1700	1600	2.10	1.46	0.2~9	compressive stress

**Table 3:** Process parameters of the ion source for depositing Ta_2_O_5_ and SiO_2_.

Material	Bias Voltage /V	Coil Current /A	Disch Voltage /V	Disch Current /mA	Ar1 Flow /sccm	Ar2 Flow /sccm	Deposition Rate /(nm/s)
Ta_2_O_5_	130	1.45	120	50	2.3	10.5	0.28
SiO_2_	160	1.8	130	55	5	7	0.7

**Table 4:** The oxygen-charging distribution scheme at the ion source (APS) and electron gun (HPE).

No.	Flow Rate of O_2_-APS/sccm	Flow Rate of O_2_-HPE/sccm
1^#^	5	45
2^#^	15	35
3^#^	25	25
4^#^	35	15
5^#^	45	5

## Data Availability

The data in this research are available from the corresponding author upon reasonable request.

## Supplementary Materials

The following supporting information can be downloaded at: https://www.mdpi.com/article/10.3390/ma16124258/s1, Figure S1: Schematic diagram of photothermal weak absorption interferometer working principle. BS1~BS3 are beam splitters, C is a chopper, L1~L4 are lenses, M1~M3 are reflectors, PM1~PM3 are power meters, PH1~PH2 are small holes, F1~F2 are bandpass filters, D1~D2 are detectors, and S is a sample; Table S1: Wide-angle depolarization reflection film and the physical thickness of each film layer.

## Abbreviations

List of symbols	
η	Optical admittance
δ	Phase of the film
Θ	Angle of incidence
τ	Phase change of the transmitted light
λ	Wavelength of electromagnetic wave
OCT	Optical coherence tomography
NIR	Near-infrared
SPTF	Stanford photothermal solutions
Ar	Argon gas
O_2_	Oxygen
USA	The United States of America
