# Supplementary material for: Development of Wide-Angle Depolarizing Reflector at 1064 nm
Source: Materials (Basel). 2023 Jun 8;16(12):4258. doi: 10.3390/ma16124258 (PMC10303084; doi:10.3390/ma16124258)
Supplement: Supplementary file 1 [file materials-16-04258-s001.zip › materials-2395624-supplementary.pdf]

Supporting information

Development of Wide-Angle Depolarizing Reflector at 1064 nm

Han Zhu [1,†], Hongyan Jiang [2,†], Kai Guo [3], Yongchao Peng [1], Yawu Xin [1], Gong Zhang [4], Yixin Lin [1], Ning Yang [1], Huashu Wei [1], Zekai Huang [1], Shifu Xiong [1,*] and Zhanggui Hu [1]

The weak absorption of thin films was measured by a photothermal weak absorption meter of model PCI-03 manufactured by SPTS, and the working principle of the equipment is shown in Figure S1. The experimental pump laser wavelength is 1064 nm with a maximum output power of 10 W, which is a stronger beam, and there is a larger thermal absorption when it passes through the sample. The detection light wavelength is 633 nm with a power of about 1 mW, which is a weak beam and (does not produce) there is not thermal absorption when it passes through the sample. The pump light is focused to the sample measurement point area after passing through the chopper with a frequency of 390 Hz. The focused spot is 20 μm in diameter, which heats the measurement area periodically with high intensity and generates a high peak "heat packet", causing a change in the optical properties of the material at the point. When the probe light passes through this region of the sample, part of the wavefront is distorted due to the change in refractive index of the material, while the other parts remain in their original state of transmission. The aberrated part of the probe light and the undistorted part produce a point diffraction common path interference, the aberration of the thermal absorption point of the material causes a change in the interference phase.

By detecting the interference field, the degree of aberration of the detection light is measured, thus the weak absorption characteristics of the sample are obtained. The detection signal of the photodetector and the reference signal generated by the chopper are fed into the lock-in amplifier simultaneously, and the signal amplitude is output after demodulation. The signal amplitude $\mathrm{S_{STL}}$ is related to the absorption of the sample test area as

$$S_{\mathrm{STL}} = \frac{I_{\mathrm{probe}} - I_{\mathrm{probe0}}}{I_{\mathrm{probe0}}} = CAI_{pump} \quad \text{(S1)}$$

Where $\mathrm{I_{probe}}$ is the average light intensity of the center of the probe spot, $\mathrm{I_{probe0}}$ is the light intensity when no "heat pack", $\mathrm{I_{pump}}$ is the pump light power, A is the absorption rate, C is a constant, and the C value is same under the same experimental conditions.

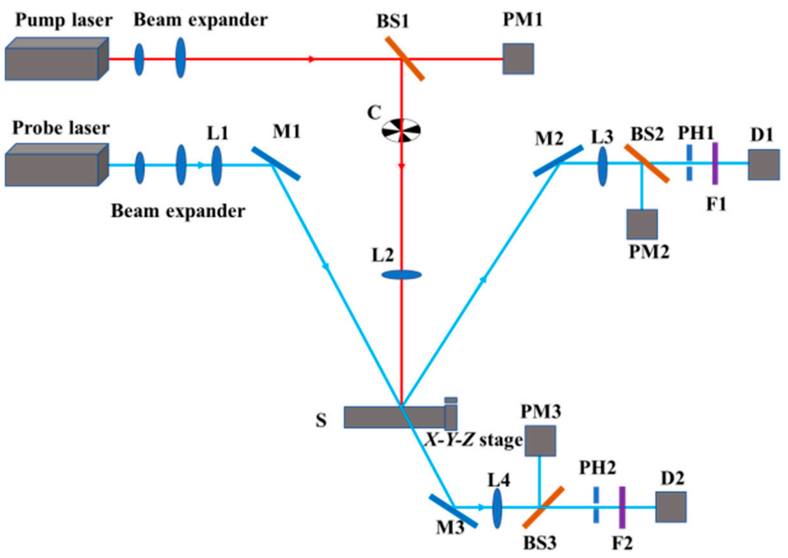

Figure S1. Schematic diagram of photothermal weak absorption interferometer working principle. BS1~BS3 are beam splitters, C is chopper, L1~L4 are lenses, M1~M3 are reflectors, PM1~PM3 are power meters, PH1~PH2 are small holes, F1~F2 are bandpass filters, D1~D2 are detectors and S is a sample.

Table S1. Wide angle depolarization reflection film the physical thickness of each film layer.

Number	Materials	Physical thickness(nm)	Number	Materials	Physical thickness(nm)
1	Ta_2O_5	130.56	29	Ta_2O_5	151.11
2	SiO_2	186.06	30	SiO_2	221.99
3	Ta_2O_5	134.13	31	Ta_2O_5	140.72
4	SiO_2	183.01	32	SiO_2	204.38
5	Ta_2O_5	132.04	33	Ta_2O_5	140.46
6	SiO_2	173.61	34	SiO_2	209.99
7	Ta_2O_5	125.91	35	Ta_2O_5	143.33
8	SiO_2	165.33	36	SiO_2	247.79
9	Ta_2O_5	128.83	37	Ta_2O_5	149.23
10	SiO_2	174.00	38	SiO_2	227.23
11	Ta_2O_5	136.31	39	Ta_2O_5	140.80
12	SiO_2	180.51	40	SiO_2	208.92
13	Ta_2O_5	139.10	41	Ta_2O_5	140.49
14	SiO_2	181.75	42	SiO_2	217.76
15	Ta_2O_5	139.19	43	Ta_2O_5	145.75
16	SiO_2	179.47	44	SiO_2	260.13
17	Ta_2O_5	136.75	45	Ta_2O_5	146.16
18	SiO_2	172.30	46	SiO_2	220.21
19	Ta_2O_5	131.57	47	Ta_2O_5	140.57
20	SiO_2	166.48	48	SiO_2	211.44
21	Ta_2O_5	131.78	49	Ta_2O_5	141.86
22	SiO_2	175.66	50	SiO_2	239.22
23	Ta_2O_5	136.99	51	Ta_2O_5	154.64
24	SiO_2	186.73	52	SiO_2	246.33
25	Ta_2O_5	140.11	53	Ta_2O_5	141.67
26	SiO_2	200.39	54	SiO_2	216.49
27	Ta_2O_5	144.42	55	Ta_2O_5	135.01
28	SiO_2	251.03			
